# Investigating Associations between Polygenic Risk Scores for Alzheimer's disease and Structural Brain Changes

**DOI:** 10.1002/alz70856_098193

**Published:** 2025-12-24

**Authors:** Snehal Pandya, Matteo De Marco, Annalena Venneri

**Affiliations:** ^1^ Brunel University London, London, United Kingdom; ^2^ University of Parma, Parma, Italy, Italy

## Abstract

**Background:**

Sporadic Alzheimer's disease (AD) accounts for >90% of AD cases, of which 70% are thought to be due to a combination of several risk genes. Investigating polygenic risk scores (PRS) that examine many genes simultaneously may increase understanding of gene‐gene interactions and their contribution to AD‐related changes in brain structure. The aim of this study was to investigate associations between PRS for AD and regional grey matter volume.

**Method:**

Data from 738 ADNI participants were examined. Two sets of PRSs were constructed, PRSwithAPOE vs. PRSwithoutAPOE, using 3 thresholds (Threshold 1: 172 SNPs including APOE vs. 121 SNPs without APOE; Threshold 2: 1561 SNPs with APOE vs. 1465 without APOE; Threshold 3: 455028 SNPs with APOE vs. 454638 SNPs without APOE). MatLab and Statistical Parametric Mapping 12 were used to process T1‐weighted brain images and to extract volumes for 114 regions of interest.

**Result:**

Multiple hierarchical linear regression models that removed the effect of demographic and clinical confounding variables showed that: (1) at whole‐group level, all PRSwithAPOE thresholds were associated with bilateral hippocampi and amygdalae volumes, R>L; (2) when stratified by diagnostic group, PRSwithAPOE Threshold 1 & 3 were associated with R hippocampus volume in cognitively unimpaired (CU) participants, PRSwithAPOE Threshold 3 was associated with the volume of the R subcallosal area in MCI, and PRSwithAPOE Thresholds 1 & 2 were associated with the volume of the L entorhinal area in AD; (3) when stratified by amyloid status, all PRSwithAPOE thresholds were associated with the volumes of bilateral hippocampi and amygdalae, R>L, and R middle occipital gyrus for amyloid positive participants. No other associations survived corrections for multiple comparisons (false discovery rate).

**Conclusion:**

PRS for AD is associated with regional grey matter volume that is characteristic of AD and is influenced by either the combination of several risk genes plus APOE SNPs or APOE SNPs alone. Association between PRSwithAPOE and volume of the L entorhinal area may distinguish AD patients from MCI/CU. Therefore, PRS may be a useful contributor to AD diagnosis.

